# Long-Chain Polyunsaturated Fatty Acids in Inborn Errors of Metabolism

**DOI:** 10.3390/nu2090965

**Published:** 2010-09-15

**Authors:** Katalin Fekete, Tamás Decsi

**Affiliations:** Department of Pediatrics, University of Pécs, József A. u. 7., H-7623 Pécs, Hungary; Email: katalin.fekete@kk.pte.hu

**Keywords:** inborn errors of metabolism, long-chain polyunsaturated fatty acids, linoleic acid, alpha-linolenic acid, arachidonic acid, docosahexaenoic acid

## Abstract

The treatment of children with inborn errors of metabolism (IEM) is mainly based on restricted dietary intake of protein-containing foods. However, dietary protein restriction may not only reduce amino acid intake, but may be associated with low intake of polyunsaturated fatty acids as well. This review focuses on the consequences of dietary restriction in IEM on the bioavailability of long-chain polyunsaturated fatty acids (LCPUFAs) and on the attempts to ameliorate these consequences. We were able to identify during a literature search 10 observational studies investigating LCPUFA status in patients with IEM and six randomized controlled trials (RCTs) reporting effect of LCPUFA supplementation to the diet of children with IEM. Decreased LCPUFA status, in particular decreased docosahexaenoic acid (DHA) status, has been found in patients suffering from IEM based on the evidence of observational studies. LCPUFA supplementation effectively improves DHA status without detectable adverse reactions. Further research should focus on functional outcomes of LCPUFA supplementation in children with IEM.

## Abbreviations

AA: arachidonic acid
ALA: α-linolenic acid
DHA: docosahexaenoic acid
E: erythrocyte
EPA: eicosapentaenoic acid
EPC: erythrocyte phosphatidylcholine
EPEA: erythrocyte phosphatidylethanolamine
EPL: erythrocyte phospholipid
HPA: hyperphenylalaninemi
LA: linoleic acid
MMA: methylmalonic academia
MSUD: maple syrup urine disease
P: plasma
PA: propionic acidemia
PCE: plasma cholesteryl ester
PKU: phenylketonuria
PPL: plasma phospholipid
PTG: plasma triacylglycerol

## 1. Introduction

Inborn errors of metabolism (IEM) represent a highly heterogeneous group of genetic conditions, in which single gene defects are responsible for a block in the metabolic pathway. The block can either be caused by the loss of function of mutant enzymes or defects of transporters. Pathological consequences are due to direct toxicity of accumulating substrates before the block, deficiency of products beyond the block, activation of alternative metabolic pathways leading to alternative metabolite production, or a combination of these factors [1]. Most IEM are autosomal recessive disorders; however, some of them are inherited in an X-linked recessive manner. Though many diseases caused by IEM are individually rare (some of them less than 1 per 100,000 births); their cumulative incidence may approach 1 per 800 to 2,500 births [2], representing thereby a serious health concern.

Neonates with IEM usually appear normal at birth, because toxic metabolites can cross the placenta and can be eliminated by the mothers. The first clinical symptoms may appear within the time interval of hours to months after birth; however, diagnoses established in childhood or even in adulthood are not exceptional [3]. Common symptoms are lethargy, poor feeding, vomiting, respiratory distress, seizures and psychomotor or developmental delay [3,4]. However, today several IEMs can be diagnosed early by neonatal screening and treatment can be initiated pre-symptomatically.

Symptoms of acute encephalopathy are particularly characteristic to patients with organic acidemias (e.g., maple syrup urine disease, propionic, isovaleric, and methylmalonic acidemias), urea cycle defects (e.g., ornithine transcarbamylase deficiency, citrullinemia) and some other disorders of amino acid metabolism (e.g., glutaric acidemia type I) [5]. Early dietary treatment with strict limitations of specific nutrient intake, together with rapid removal of toxic substrates and/or replacement of the deficient products are the main therapeutic measures securing long-term survival of these patients [6,7]. Patients with severe IEMs have to avoid not only foods of animal origin, but usually also some plant-based foods with high protein contents. For instance, patients with severe hyperphenylalaninemia can usually tolerate only 200–400 mg phenylalanine intake, which equals to 120–240 mL of cow’s milk or 80–160 g of beans per day. The severe dietary protein restriction can be compensated by consuming special amino acid mixtures that are devoid of the critical amino acids. Still, dietary protein restriction may lead both to reduced amino acid intake and to low intake of important other nutrients, such as vitamins, trace elements, and polyunsaturated fatty acids as well [8,9]. This review discusses the potential consequences of reduced bioavailability of long-chain polyunsaturated fatty acids (LCPUFAs).

## 2. Long-Chain Polyunsaturated Fatty Acids

LCPUFAs are important components of membrane lipids in all tissues. The most important of them are the omega-6 essential fatty acid, linoleic acid (C18:2n-6, LA), and the omega-3 essential fatty acid, α-linolenic acid (C18:3n-3, ALA), as well as their longer-chain metabolites, arachidonic acid (C20:4n-6, AA) and docosahexaenoic acid (C22:6n-3, DHA). LCPUFAs increase the fluidity, flexibility and permeability of cell membranes, the number of receptors and the affinity of receptors to their substrates: hormones, growth factors, and proteins. Moreover, some LCPUFAs are also precursors of several second messengers. Omega-6 fatty acids, mainly AA and dihomo-γ-linolenic acid (C20:3n-6), are predominantly precursors of proinflammatoric prostaglandins, thromboxans and leucotriens, while omega-3 fatty acids, mainly eicosapentaenoic acid (C20:5n-3, EPA), are precursors of antiinflammatory eicosanoids. AA and DHA are concentrated in the central nervous system, as well as in the retina, heart and skeletal muscle, and play an important role in the maintenance of normal development and normal neural functions [10].

**Figure 1:**
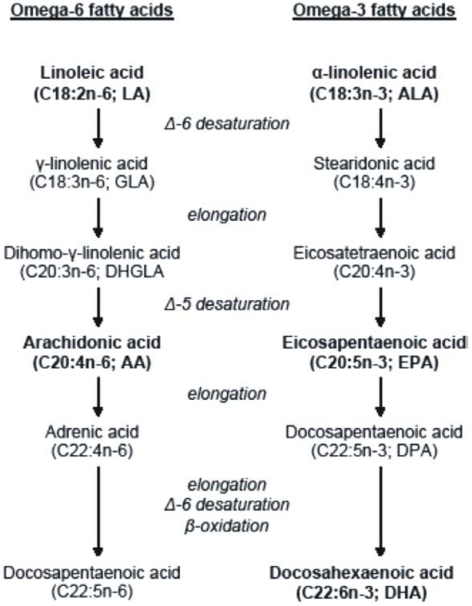
Metabolism of the omega-6 and omega-3 fatty acids.

Vegetables are good sources of essential fatty acids; however, their preformed long-chain metabolites are found mainly in animal foods. Food products of terrestrial animals are rich in omega-6 fatty acids, whereas sea fishes are rich in omega-3 fatty acids [11]. Mammals, including humans, cannot synthesize essential fatty acids; therefore they have to consume them in the diet from dietary sources. The enzymatic reactions of Δ-6- and Δ-5 desaturation and elongation of essential fatty acids convert LA to AA and ALA to EPA. While AA is the major product of the omega-6 fatty acid family, EPA is an intermediate, which needs further elongation, Δ-6 desaturation and peroxysomal β-oxidation to be converted into the biologically most important product, DHA (Figure 1) [12].

The conversion of LA to AA is more efficient than the conversion of ALA to DHA, because AA synthesis is less complex than DHA synthesis. In healthy individuals, the conversion rate of ALA to EPA is less than 5 to 10% and to DHA is only 2 to 5% [13]. Because the elongation reactions are rapid, desaturation reactions represent the rate-limiting steps [11]. Moreover, essential fatty acid content of the diet can also influence the conversion rates; because the involvement of the same enzymes in the synthesis of the two fatty acid families leads to strong metabolic competition between omega-6 and omega-3 fatty acids. Due to the relatively limited effectiveness of endogenous DHA synthesis, the DHA status is determined dominantly by dietary intake of preformed DHA [14]. It has been demonstrated in numerous studies that vegans and vegetarians, who have diets high in LA, have a lower omega-3 LCPUFA status than omnivores (lower EPA and DHA) [15].

## 3. Literature Search

We conducted a MEDLINE literature search using the PubMed as well as OVID interface; Scopus and the Cochrane Library CENTRAL databases were also searched from inception to May 2010. Text terms with appropriate truncation and indexing terms were used to identify articles eligible for review. The search was in the following form: [(IEM terms) AND (LCPUFA terms)]. The searches were supplemented with hand searches of journals and the reference lists from relevant articles located were used to identify additional sources. We did not apply any language restriction. 

## 4. Long-Chain Polyunsaturated Fatty Acids in Inborn Errors of Metabolism

Despite the early initiation of treatment, patients with IEM usually suffer from long-term neurological complications; mainly brain structural changes and abnormalities of the visual function have been shown in patients who are otherwise well-controlled during childhood, e.g., patients suffering from hyperphenylalaninemia [16]. There is considerable interest in the potential roles that LCPUFAs play in neurological development throughout childhood. It seems possible that LCPUFA deficiency induced by the dietary treatment may also contribute to neurological abnormalities. 

During fetal life, LCPUFAs are provided by placental transfer; thereafter, these fatty acids are provided either in breast milk or in infant formulas. Human milk is a relatively low-protein food, it contains LCPUFAs and many other bioactive compounds; nevertheless, breastfeeding contributes only a relatively small extent to the nutrition of infants with IEM [17,18]. Because of the limited dietary intake of preformed omega-6 and omega-3 LCPUFAs, many patients with IEM must rely on the endogenous synthesis of LCPUFAs from their precursors, LA and ALA [14]. 

### 4.1. Observational studies

Fatty acid status has been investigated in several groups of patients with different IEM (Table 1). The majority of studies were carried out in PKU children, but patients with other inborn errors of amino acid metabolism were also investigated. Numerous lipid classes were analyzed in order to reveal potential differences between fatty acid profiles of patients with IEM and healthy, age-matched controls. In five studies, AA levels did not differ in patients and controls [19,20,21,22,23]; this finding indicates that most patients investigated in these studies can maintain normal AA status, if LA intake is adequate. However, in four studies, patients with IEM have been found to have significantly lower plasma AA levels than that of the controls, in spite of equal [24,25] or even significantly higher [26,27] values of the precursor essential fatty acid, LA. Even more marked reduction was observed in DHA status in patients with IEM. DHA levels were significantly lower in patients with IEM compared to healthy controls in eight studies, whereas in two studies [19,21], DHA levels did not differ between patients and controls. The most important intermediate metabolite of DHA synthesis, EPA, showed significantly lower levels in patients than in controls in eight studies, whereas no differences were seen in two other studies [19,23]. No clear picture of the availability of the precursor omega-3 essential fatty acid, ALA was seen in the studies reviewed (Table 1).

**Table 1:** Observational studies on the availability of long-chain polyunsaturated fatty acids in inborn errors of metabolism. Number of participants in the IEM* group: methylmalonic acidemia (5), ornithine transcarbamylase deficiency (7), citrullinemia (1); number of participants in the IEM** group: ornithine transcarbamylase deficency (7), argininosuccinic aciduria (4), methylmalonic acidemia (6), PA (3), MSUD (1), tyrosinemia type I (5), classical homocystinuria (4), lysinuric protein intolerance (1), 2-amino-/2-oxoadipic aciduria (1), hyperinsulinaemia–hyperammonaemia syndrome (1); medical food for patients:^a^: Phenex-1, -2; ^b^: Phenyl-Free; ^c^: XP Maxamaid/Maxamum. n.d.: no data; ↑: patients had significantly higher values (p < 0.05) than healthy controls; ↓: patients had significantly lower values (p < 0.05) than healthy controls; —: no significant difference between patients and healthy controls.

Study	Number of participants, age	Biomarker	LA	AA	ALA	EPA	DHA
Galli *et al.*, 1991 [24]	PKU(15)–Control(12) 3–12 yr	P	—	↓	n.d.	↓	↓
		PPL	—	↓	n.d.	—	↓
		PCE	—	—	n.d.	n.d.	n.d.
		E	—	—	n.d.	—	—
Sanjurjo *et al.*, 1994 [28]	PKU(40)–Control(50) 2 mo–20 yr	P	↑	↓	—	—	↓
		EPL	—	↑	↓	↓	↓
Sanjurjo *et al.*, 1997 [26]	IEM*(13)–Control(50) 1–17 yr	P	↑	↓	↓	↓	↓
		EPL	↑	—	↓	—	↓
Decsi *et al.*, 1997 [19]	PA(5)–Control(18) 3.5–9.5 yr	PPL	—	—	—	—	—
		PTG	—	—	—	—	—
		PCE	—	—	—	—	—
Pöge *et al.*, 1998 [20]	PKU(8)–Control(12) ^1^ PKU(9)–Control(8) ^2^; ^1^: 1–6 yr; ^2^: 11–18 yr	PPL	—	—	—	—	—
			—	—	—	—	—
		PCE	—	—	—	—	↓
			—	—	—	—	—
		EPC	—	—	—	—	↓
			—	—	—	—	—
		EPEA	—	—	—	↓	↓
			—	—	—	—	—
van Gool *et al.*, 2000 [27]	PKU(9)–Control(18) 6 mo–25 yr	PPL	—	—	↓	↓	↓
		EPL	↑	↓	↓	↓	↓
Acosta *et al.*, 2001 [21]	PKU(13)–Control(13) ^a^ PKU(7)–Control(6) ^b^ PKU(8)–Control(7) ^c^ 1–13 yr	P	—	—	↑	—	—
			—	—	—	↓	—
			—	—	—	—	—
		E	↑	—	—	—	—
			—	—	—	—	—
			—	—	—	—	—
Moseley *et al.*, 2002 [25]	PKU(27)–Control(120) 7–39 yr	P	—	↓	↑	↓	↓
		E	—	—	—	↓	↓
Vlaardingerbroek *et al.*, 2006 [22]	IEM**(33)–Control(38) 1–18 yr	PPL	↑	—	↑	↓	↓
		EPL	—	—	—	↓	↓
Mazer *et al.*, 2010 [23]	MSUD(6)–Control(12) 12–30 yr	P	—	—	↑	—	↓
		E	—	—	↑	—	↓

### 4.2. Randomized controlled trials

We were able to identify six randomized controlled trials (RCTs) on the effect of LCPUFA supplementation in patients with IEM (Table 2). In two studies, infants with PKU consumed phenylalanine-free infant formula with or without LCPUFA for one year [29,30]. At the end of the intervention, DHA levels were significantly higher in supplemented than in control infants, whereas AA levels did not differ between the groups. Neither in visual evoked potentials, nor in mental and psychomotor development indices (Bayley Test) were differences reported between supplemented and control infants in one of these studies [29].

In the other four RCTs, children either with PKU (three studies) or with MMA (one study) were supplemented with LCPUFA. In a single blind placebo controlled trial, children with PKU were randomized to fish oil or blackcurrant oil supplementation [31]. After six months of dietary intervention, subjects receiving fish oil showed significantly higher EPA and DHA values than controls; moreover, significantly decreased plasma triacylglycerol values was observed in the fish oil group. In a double blind, placebo controlled trial children with PKU received capsules containing either equivalent amounts of omega-6 and omega-3 LCPUFAs or placebo. DHA levels in the supplemented group increased by around 100%; whereas AA and EPA level remained almost the same [16,32,33]. By the end of the study, P100 wave latency decreased significantly in the supplemented group; however, P100 wave latency returned to the baseline after three years [33]. In another open-labelled, randomized supplementation trial [34], children with PKU received fat-free protein substitute with or without essential fatty acids for 20 weeks. DHA level increased significantly; however AA levels did not increase over the study period. In an open-labeled, randomized study of crossover design, children with MMA received DHA treatment or placebo [35]. Plasma DHA level increased and triacylglycerol levels decreased significantly with DHA therapy. It is to be emphasized that none of the studies summarized in Table 2 reported any adverse reactions to LCPUFA supplementation.

**Table 2:** Randomized controlled trials on the effect of long-chain polyunsaturated fatty acid supplementation in inborn errors of metabolism. *: % DHA/total fatty acid composition; a: mean (SD); b: mean (range); ↑: significant increase (p < 0.05) in the supplemented group compared with the control group at the end of the intervention; ↓: significant decrease (p < 0.05) in the supplemented group compared with the control group at the end of the intervention; —: no significant difference between the treatment and the control group at the end of the intervention.

Study	Number of participants; age	Short description of intervention	Biomarker	DHA * (treatment *vs.* control group)	Clinical outcomes
[29]	42 infants with PKU (21 in treatment and 21 in control group); 8–39 days	Supplemented formula (0.7 g AA and 0.3 g DHA/100 g fatty acids) for 1 yr	EPL	3.60 (1.06) *vs.* 1.40 (0.44) ^a↑^	— P1 and P100 latency; — mental and physical development (Bayley Test); no adverse reactions
[30]	21 infants with PKU (10 in treatment and 11 in control group); <4 wk	Supplemented formula (0.46 g AA and 0.27 g DHA/100 g fatty acids) for 1 yr	PPL	3.08 (0.10) *vs.* 1.52 (0.19) ^a↑^	no adverse reactions
[31]	21 children with PKU (10 in treatment and 11 in control group); 5–10 yr	2.5–4 g fish oil (18 g EPA, 4 g DPA and 12 g DHA/100 g fatty acid) daily for 6 mo	P	2.94 (0.88) *vs.* 0.73 (0.08) ^a↑^	↓ plasma triacylglycerol; no adverse reactions
[16,32,33]	20 children with HPA (10 in treatment and 10 in control group); 10 ± 7 yr	1 capsule (37 mg AA, 27.5 mg EPA, 20 mg DPA and 40 mg DHA/0.5 g capsule) per 4 kg body weight for 1 yr	P	2.3 (1.1) *vs.* 1.1 (0.3) ^a↑^	↓ P100 wave latency; — plasma triacylglycerol; no adverse reactions
			PPL	3.1 (1.6) *vs.* 1.6 (0.4) ^a↑^	
			PTG	0.6 (0.5) *vs.* 0.3 (0.3) ^a—^	
			PCE	0.5 (0.2) *vs.* 0.2 (0.1) ^a↑^	
			E	2.8 (1.5) *vs.* 1.5 (0.5) ^a—^	
			EPC	0.9 (0.3) *vs.* 0.4 (0.2) ^a↑^	
			EPEA	3.7 (1.7) *vs.* 1.3 (0.9) ^a↑^	
[34]	44 children with PKU (24 in treatment and 20 in control group); 1–10 yr	EFA supplemented protein substitute (17.2 g LA and 4.5 g ALA/100 g fatty acid) for 20 wk	EPL	2.07 (0.8) *vs.* 1.64 (0.4) ^a↑^	no adverse reactions
[35]	4 children with MMA; 9–16 yr; crossover design	25 mg/kg DHA daily for 3 mo	P	5.14 (2.72–7.94) *vs.* 1.89 (1.12–2.31) ^b↑^	↓ plasma triacylglycerol; no adverse reactions

## 5. Conclusions

From observation trials there is convincing evidence indicating that patients suffering from IEM have lower contribution of LCPUFA, especially of DHA, to the fatty acid composition of various plasma and erythrocyte membrane lipids than healthy controls. From RCTs there is firm evidence showing that LCPUFA supplementation results in higher DHA status without detectable adverse reactions. The limited data available on functional consequences of LCPUFA supplementation in children with IEM do not allow any firm conclusion to be drawn as yet. Further research should focus on functional outcomes of LCPUFA supplementation in children with IEM.
